# Nurses’ experiences of caring for patients with intellectual developmental disorders: a systematic review using a meta-ethnographic approach

**DOI:** 10.1186/s12912-018-0316-9

**Published:** 2018-12-03

**Authors:** Marie Appelgren, Christel Bahtsevani, Karin Persson, Gunilla Borglin

**Affiliations:** 1 Department of Care Science, Faculty of Health and Society, Malmö University, SE-205 06 Malmö, Sweden; 2 City of Malmö, Borough Administration Operation Support Management, SE-205 80 Malmö, Sweden

**Keywords:** Care, Idiomatic translations, Intellectual disability disorder, Line of argument synthesis, Qualitative research, Qualitative synthesis

## Abstract

**Background:**

Research suggests that registered nurses (RNs) do not feel adequately prepared to support patients with intellectual disability disorder (IDD). This is unsurprising, as few European health sciences curricula include undergraduate and graduate training courses in IDD. As RNs are often in the front line of care, eliciting in-depth knowledge about how they experience nursing this group of patients is vital. Our aim in this study was to develop a conceptual understanding about RNs’ experiences of nursing patients with IDD.

**Method:**

We undertook a systematic review and meta-ethnography to synthesise qualitative research studies found in PubMed, CINAHL, PsycINFO, ERIC databases and by manual searching to identify additional studies. We condensed translatable second-order constructs, and developed an idiomatic translation. Finally, we formulated line of argument (LOA) syntheses to capture the core of the idiomatic translations.

**Results:**

We included eighteen published studies from eight countries involving 190 RNs. The RNs’ experience of nursing patients with IDD were reflected in 14 LOAs. Six of these reflected a tentatively more distinctive and at times unique conceptualisation of RNs’ experience of nursing this group of patients. The remaining eight LOAs represented a conceptualisation of nursing per se, a conceptualisation of nursing that was interpreted as a universal experience regardless of context and patient group.

**Conclusion:**

Lack of awareness and knowledge are likely breeding grounds for the ‘otherness’ that still surrounds this group of patients. In encounters between patients and RNs, focusing on the person behind the disability label could be one way to secure relevant nursing care for patients with IDD. Undertaking appropriate under- and postgraduate education alongside the implementation of nursing models focusing on patient-centred care would help RNs in reducing the health and care inequalities this group of patients still face.

**Trial registration:**

PROSPERO 2017: CRD42017077703.

**Electronic supplementary material:**

The online version of this article (10.1186/s12912-018-0316-9) contains supplementary material, which is available to authorized users.

## Background

Promoting the health of patients with intellectual developmental disorder (IDD) should be of universal concern to all nurses. People with disabilities have the same right to the highest attainable standard of health as everybody else [1]. Despite this, there seems to be little discussion about their actual health needs. How registered nurses (RNs) should be educationally prepared to support this group of patients and what role they should play in addressing their health needs is under-researched in the international nursing literature [2]. Additionally, the bulk of research seems to have focused on parenting children with IDD [3], parents with IDD [4], health and social care for those with a dual diagnosis, for example, IDD and somatic health problems [5], challenging behaviour (CB) associated with IDD [6, 7] and burn out among frontline carers of people with IDD [8]. Therefore, research investigating how RNs experience nursing, for this vulnerable group of patients is vital for knowledge development within nursing across health care settings.

One of the major challenges in nursing patients with IDD appears to be that RNs in general health care settings are not adequately prepared to support their health needs. This is also true in the acute care settings [9]. Because these patients are not viewed as a distinct and vulnerable group with health care needs outside those of the general population, the necessary education and training for health care staff has not been put into place [10]. In these circumstances, it is highly likely that in health care staff will not understand specific issues relating to IDD or that reasonable adjustments in care are needed for this patient group. Melville and colleagues [11] have identified a clear knowledge gap among health care practitioners with only 8% of the RNs in their study receiving learning disability-related training. In their systematic review, Bradbury-Jones and colleagues [12] highlighted health care professionals’ limited knowledge about IDD and concluded that patients with IDD are misunderstood in care. The literature also reports that there are international disparities in educational levels for health and social work staff. It appears reasonable to propose that a lack of preparation and knowledge is the most likely explanation for these reported problematic attitudes and negative behaviour among health care professionals in relation to this patient group [13, 14]. For example, in a study by Lewis and Stenfert-Kroeses [15], nursing staff at a general hospital reported less positive attitudes concerning caring for patients with IDD compared to caring for patients with somatic disorders. Indifference and negative attitudes can lead to negative consequences in terms of quality of care for this patient group as evident in a recent Mencap report [16] and in the Confidential Inquiry into premature deaths of people with learning disabilities, CIPOLD [17] from the United Kingdom (UK). Such negative behaviour and attitudes, together with a lack of training in good practice is likely to negatively affect the quality of nursing offered to this patient group. This is particularly distressing as these patients experience the same range of health problems as others but, according to Cooper and colleagues, [18] with an increased risk of co- or multimorbidity in comparison to the wider population. However, the pattern of illness they experience may be different [19], leading to increased frailty that predisposes them to an earlier burden of disease [20]. According to Campbell [21], patients with IDD have more complex health needs than the general population but face much greater difficulty in getting good and adequate health care. This is supported by Brown and colleagues [22], who state there is evidence that these patients’ needs are often poorly meet by health services, with many experiencing significant barriers to accessing health care appropriate for their individual requirements. This is further underlined by Heslop et al., [17] who in their investigation into premature deaths of people with intellectual disability in the UK, found that problems with the provision of care e.g. problems in advanced health and care planning, including recognising needs and adjusting care as needs change, together with problems with service provision e.g. delays in the diagnosis and treatment of health care problems, contributed to deaths that were significantly different for a subset of people with intellectual disabilities and comparator cases. Registered Nurses therefore should have an important role in caring for patients with IDD, particularly as they are expected to lead nursing care and to support health care assistants and/or assistant practitioners in caring for this growing group of people.

In the past 40 years, developed countries have seen far-reaching and radical changes in peoples’ attitudes towards people with IDD. A growing emphasis on the provision of services in the community has led to the closure of long-stay hospitals and institutions, with greater inclusion in wider society [23]. An increase in health screening and medical interventions has resulted in a greater awareness that this group of people will live longer. According to Ervin and Merrick [24], the number of adults with IDD aged 60 years and older is expected to reach 1.2 million by 2030. However, according to Innes, McCabe and Watchman [25] there is no corresponding evidence regarding how support will and should be provided. It is therefore probable that RNs, regardless of in which health care setting they work, will encounter this patient group on a more regular basis, presenting a challenge for the health care professionals who need to provide them with care in a timely and relevant manner. Therefore, it is particularly noteworthy that the majority of European countries, with the exception of the United Kingdom and Ireland, provide few undergraduate and graduate training courses in IDD within their health sciences curricula. Therefore, not all European patients with IDD will meet RNs with the relevant competence and skills needed to offer adequate nursing to these patients. Consequently, knowledge on how to organise and deliver optimal and safe evidence-based care to this population is of huge importance. Some good practice models with regard to the nursing of this patient group for example, hospital passports, legislations on reasonable adjustments, health action plans and hospital liaison RNs, are evident, especially in the UK but the evidence indicates there is still some way to go to embed these practices elsewhere. As RNs are often on the front line of both primary and secondary care, it seems natural that as a profession nurses engage in knowledge development within this field, particularly as the evidence base for IDD nursing practice remains limited in both quantity and quality [26]. Therefore, the overall aim of this systematic literature review is to develop a conceptual understanding of RNs’ experiences of nursing patients with IDD.

## Method

The design of this meta-synthesis was influenced by the meta-ethnographic method described by Noblit and Hare [27]. The growing use of this method in health services research [28] means the method has evolved. Meta-ethnography is an interpretive rather than an aggregate form of knowledge synthesis and accordingly aims to develop conceptual understandings—in this case about RNs’ experience of nursing patients with IDD—rather than to provide an aggregate account of findings [27]. Our design is equally informed by other more recent methodological accounts [29, 30].

### Search strategy

We designed our search strategy, overall study design and structure for this meta-synthesis in accordance with the Preferred Reporting Items for Systematic Reviews and Meta-analyses Statement for Protocols (PRISMA-P) [31] (Additional file 1). We developed our search strategy using a modified form [32] of the SPIDER tool developed by Cook and colleagues [33], including terms for sample, phenomenon of interest and research design (Table 1). We conducted an initial scoping search to gain an understanding about how studies might be indexed and to find terms used in titles and abstracts. This initial search was also done to assess the suitability of the review questions and to understand what literature volumes to expect [34]. Our initial scoping search indicated we would not end up with an unreasonable number of papers; therefore, we set no limitations in terms of dates for our searches.

**Table 1:** Breakdown of the research question

SPIDER heading	Search topics
S - sample	Nurses
PI – phenomenon of interest	Nursing for persons with intellectual disability disorders
D & R – design and research type	Qualitative research

We initially searched PubMed, CINAHL, PsycINFO and ERIC in November 2016 (Additional file 2). Due to a time lapse between the search and the synthesis, we conducted a repeat search just before work on the synthesis began (November 2017) to ensure all relevant papers were included. We identified no additional studies of relevance. We used free-text terms to access studies not yet indexed and database-specific subject headings. To identify pertinent search terms in the databases, we transformed terms into medical subject heading (MeSH) terms and/or headings (e.g. intellectual developmental disorder, nursing, experiences, qualitative research). We combined search terms with the Boolean operator OR to make the search sensitive and then with the Boolean operator AND to assure specificity within the search strategy [35].

### Selection of studies

We included studies if they (i) were in English, (ii) were published in a peer-reviewed journal, (iii) used qualitative methods and demonstrated qualitative analysis and (iv) reported the experiences of nursing for adults with IDD regardless of health care context and the RNs type of education. We used the ICD (International Classification of Diseases) 11 definition of IDD, which is ‘a group of conditions characterised by significant impairment of cognitive functions, which are associated with limitations of learning, adaptive behaviour and skills’ [36]. We used the International Council of Nurses (ICN) definition of nursing, which is the ‘autonomous and collaborative care of individuals of all ages, sick or well and in all settings. Nursing includes the promotion of health, prevention of illness, and the care of ill, disabled and dying people’ [37]. Our decision to include any type of qualitative design was based on the assumption that regardless of individual methodology, qualitative designs overall derive from the same epistemological and ontological perspectives.

We excluded studies if: (i) they used mixed methods where qualitative data could not be separated out; (ii) data analysis was lacking the necessary conceptual depth, that is, we assessed it as only containing non-translatable concepts [29]; (iii) data from RNs could not be distinguished from those of other health care professionals or if informants could not be identified as RNs; (iv) data were derived from open-ended questions in surveys.

**Fig. 1:**
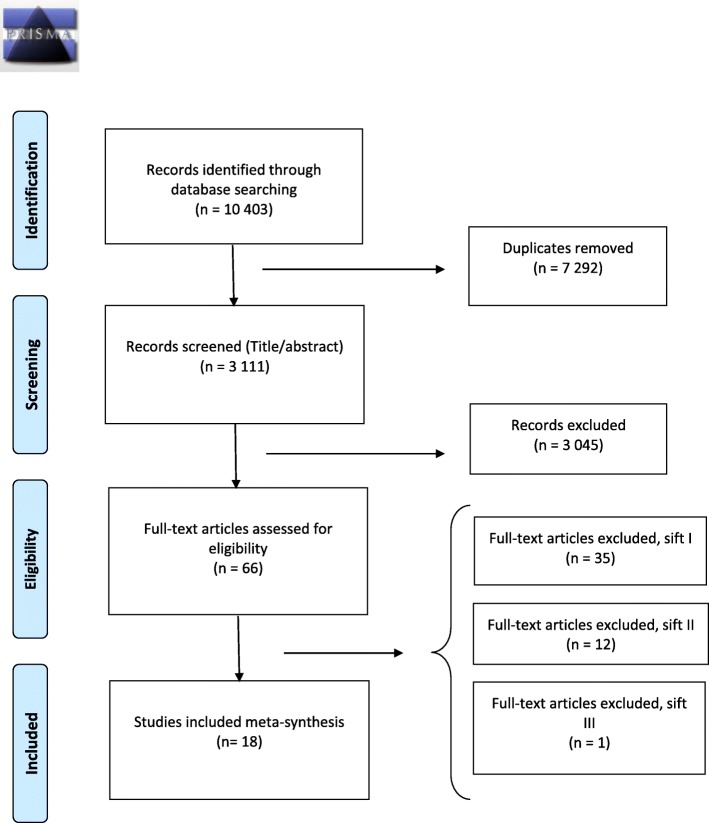
Flow chart literature search

The first author (MA) screened the 3111 studies by title and abstract, resulting in the exclusion of 3045 studies. Following the application of the inclusion and exclusion criteria to the 66 studies read in full text, a further 35 (Additional file 3) studies were excluded (Sift I), leaving 31 studies assessed as eligible for inclusion (Fig. 1). No further studies were located via a manual search of reference lists.

### Data extraction

The first author (MA) critically appraised and extracted data from all included studies after Sift I from all 31 studies. Two of the co-authors (CB and KP) acted as second reviewers of 10 studies each, while the third co-author (GB) acted as second reviewer of 11 studies.

We developed our appraisal and data extraction protocols based on the ideas of Toye et al. [29] and Schütz [38] regarding first- and second-order constructs commonly used in meta-ethnographic studies to distinguish the data [29]. Next, we extracted data relating to study design, nursing focus, IDD focus, context, sample size and demographics (gender, education, years of practice), methodology, data collection and data analysis methods and clearly articulated second-order constructs, such as concepts, metaphors, themes and/or categories, (a copy of the data extraction protocol can be obtained from the authors). To be included in the meta-synthesis of this review, concepts needed to go beyond description alone, in accordance with Toye and colleagues [29]. This means that concepts should explain the data, that is, to be translatable. We consequently excluded all second-order concepts independently assessed by both reviewers as non-translatable. Schütz [38] makes a clear distinction between first-order constructs, that is, the participants’ interpretation of their own words, and second-order constructs, that is, the researchers’ interpretation of the participants’ first-order constructs. We extracted second-order constructs relevant for our review question together with underpinning text and later each pair of reviewers read and assessed them as either translatable or non-translatable second-order concepts.

### Quality appraisal

We conducted a methodological quality appraisal of the studies, following the conceptual model for quality appraisal of Toye et al. [29]. Their model suggests two core facets of quality for inclusion in meta-ethnography, as follows:

1. Conceptual clarity: How clearly has the author(s) articulated a concept that facilitates theoretical insight?
2. Interpretive rigour: What is the context of the interpretation? How inductive are the findings? Has the interpretation been challenged?

In accordance with Toye et al. [29], we categorised each study as either a key paper (KP), a satisfactory paper (SP), a fatally flawed paper (FFP) or an irrelevant paper (IRP). If there was any disagreement between the reviewer pairs, a third reviewer reviewed the studies, which occurred in one case. During this process (Sift II), 12 further studies were assessed by both reviewers as IRPs (Additional file 3) and were therefore excluded; this left 19 studies.

### Data translation and line of argument synthesis

Based on our developed appraisal and data extraction protocol, we transferred data into a Word document i.e. to a synthesis matrix including study identifier and translatable second-order concepts with supporting text explaining and describing the concept for each study. We created free space in the matrix for our non-linear process of condensing the supporting content into idiomatic translations focusing on salient categories of meaning rather than the literal translations of words or phrases [27]. According to Campbell and colleagues [39], it is in the translation that data becomes synthesised; thus, a full understanding of the concepts can be reached. We continuously compared the meaning—the core of all translatable second-order concepts. The first author (MA) and the last author (GB) worked in close collaboration throughout the process, and every step of the process was discussed by all the authors at meetings about twice a month. This meant that the condensations and idiomatic translations evolved in a dynamic interpretative process that helped us identify similarities and differences within and across the included studies. We then formulated a line of argument (LOA) synthesis, capturing the core of our idiomatic translation. The LOA synthesis represented those translatable concepts within and between studies, interpreted to represent the same core meaning [27], that is, an overarching conceptual understanding of the experience of nursing patients with IDD (Table 2). We thereafter re-examined and re-assessed each study for its relevance to each individual LOA synthesis and checked the relevance of our interpretations. During this process, we identified one further study as an IRP (Additional file 3), leaving 18 studies in the final synthesis (Fig. 1).

**Table 2:** Example of the synthesis process

Study no./Reference	Translatable Concept (TC)	Sub-categories	Content supporting TC	Idiomatic translation	Line of Argument synthesis
S1. Donovan, 2012. [48]	*The importance of a caring relationship with the client*	NA	*Participants indicated that an in-depth relationship with the client was important to them. This had often developed over a long period of time and enabled them to recognize the often-subtle changes in temperament or behavior that had occurred.*	Nursing people with IDD can mean having a long-term in-depth relation to be able to read and to understand the person	Based on long-term relationship **[A**^a^**]**
S26. Slevin & Sines, 2005. [52]	*Relational therapeutics*	NA	*Forming trusting relationships were considered essential. Building caring relationships was not only considered to be valuable for the client, but a holistic approach was followed in which the relationship with the total family was developed. The value placed on a humanistic approach in caring was evident. They wished to empower parents/clients and to do this they developed relationships, trust, and aligned themselves with the family.*	Nursing people with IDD can mean having an in-depth relation both with the patient and his/her relevant others in order to be able to provide a care that empower all included	Based on long-term relationship **[A**^a^**]** Rest its foundation on trust **[B]**
S21. Ndengeyingoma & Ruel, 2006. [54]	*The challenge of assuring the expected level of quality care*	Challenges relating to organizing care delivery	*Two main challenges directly affecting nursing care were identified: managing patients’ behavioral reactions and communication difficulties. Participants perceive evaluations of these patients to be incomplete. They cannot explain interventions or respond to patients’ needs as they would like. A participant exemplifies: “There is always a discomfort with a patient who has an ID; we are not used to that.*. *. I have sometimes been alone with such a client, and honestly, her reactions as I approached to take her vital signs made me feel helpless”.*	Nursing people with IDD can mean having challenges in managing the patients´ behavior and in communication	Go beyond verbal communication alone **[C**^a^**]**
S12. Morton-Nance & Schafer, 2012. [51]	*Communication*	NA	*Communication was a major barrier to understanding patients’ needs. Difficulties in communication between healthcare professionals were thought to affect the quality of palliative care. Participants raised concerns about a failure to share important information appropriately, making it difficult to meet patients’ basic needs. Patients’ inability to communicate their needs was thought by participants to exacerbate problems. Inexperience and lack of understanding, skills and training on the part of some careers significantly affected quality of care at the end of life.*	Nursing people with IDD can mean challenges in regards to communication between patients, health care professionals and relevant others. Nursing people with IDD can mean challenges in regards to the skills and abilities of HCPs which leads to unmet needs and poor quality of care	Go beyond verbal communication alone **[C**^a^**]** Raise the bar in care for patients with IDD **[G**^a^**]** *Additional perspective from the core conceptualization:* e.g. as in eliminating unmet care needs

^a^=LOA synthesis interpreted to represent a tentatively distinct conceptualization of nursing

## Results

We included eighteen studies involving 203 informants (5–27 informants per study) in this review (Table 3). About 70 of the informants were either described as being an intellectual disability or a learning disability RN, and 133 of them were described as being general RNs. Most of the studies were conducted in the United Kingdom (*n* = 9), followed by Ireland (*n* = 3) and Northern Ireland (*n* = 2). The remaining studies were conducted in Canada, Sweden, the United States and New Zeeland. Eleven studies were categorised as reporting on a qualitative study guided by an explicit set of philosophical assumptions in the form of one of the known classical qualitative designs (CQDs), while seven studies were categorised as having generic qualitative research designs (GQDs) [40].

**Table 3:** Overview included studies

Study	Study aim	Sample and context	Methodological approach	Data collection and analysis	2nd Order Concepts	LOA synthesis	Quality appraisal (Toye et al., 2013)
Blackmore, 2001, United Kingdom. **(S23**)	Explore learning disability nurses´ perceptions about their role in advocating for clients who have ID + physical, sensory and communication disabilities	*N* = 8 RNLDs Gender not known Community Trust, LD Directorate	Generic qualitative design	Semi-structured interviews Constant comparison	Translatable 2nd order concepts *n* = 2 Non-translatable 2nd order concepts *n* = 4	**[F**^a^**]** Work against negative attitudes and alienation **[M]** Entail advocacy and safe guarding	Fatally flawed paper
Boarder, 2002, United Kingdom. **(S28)**	Obtain an overview of Community Learning Disability Nurses´ perceptions of their work	*N* = 20 RNLDs Gender not known NHS Trusts in Wales	Generic qualitative design	Semi-structured interviews Content analysis	Translatable 2nd order concepts *n* = 3 Non-translatable 2nd order concepts *n* = 1	**[F**^a^**]** Work against negative attitudes and alienation **[G**^a^**]** Raise the bar in care for this patient group **[N**^a^**]** Understands the complexity of this patient group	Fatally flawed paper
Campbell, 2011, United Kingdom. **(S16)**	Describe participants’ emotional response to violence and to explore what support they require in dealing with constant exposure to workplace violence.	*N* = 6 RNs Gender not known Unit for adults with ID and challenging behaviour	Generic qualitative design	Semi-structured interviews, Thematic analysis	Translatable 2nd order concepts *n* = 2 Non-translatable 2nd order concepts = 0	**[J**^a^**]** Take unpredictable situations into account	Satisfactory paper
Donovan, 2012, United Kingdom. **(S1)**	Describe the experiences of Learning Disability Nurses when they are with clients who may be in pain but who cannot communicate their feelings verbally.	N = 8 RNIDs Gender not known Care homes [NHS Trust]	Phenomenology	Unstructured interviews	Translatable 2nd order concepts n = 2 Non-translatable 2nd order concepts *n* = 1	**A**^a^**]** Based on long-term relationships **[B]** Rest its foundation on trust **[C**^a^**]** Go beyond verbal communication alone	Fatally flawed paper
Doody et al., 2012, Northern Ireland. **(S2)**	Explore the experiences of RNIDs caring for older people with ID.	*N* = 7 RNIDs ♀ 5 + ♂ 2 Voluntary service providing community care in residential settings	Phenomenology	Semi-structured interviews Thematic analysis	Translatable 2nd order concepts *n* = 3 Non-translatable 2nd order concepts = 0	**[A**^a^**]** Based on long-term relationships **[D]** Be forward planning **[E]** Include relevant others to offer quality care **[F**^a^**]** Work against negative attitudes and alienation **[G**^a^**]** Raise the bar in care for this patient group **[H]** Acknowledge the person behind the label of disability **[I]** Based on evidence-based practice	Fatally flawed paper
Fitzgerald et al., 2013, Ireland. **(S18)**	Explore nurses´ perceptions of their role in the area of ID care	N = 7 RNs ♀ 7 Community residential service	Generic qualitative design	Semi-structured interviews Latent content analysis	Translatable 2nd order concepts *n* = 2 Non-translatable 2nd order concepts *n* = 2	**[A**^a^**]** Based on long-term relationships **[C**^a^**]** Go beyond verbal communication alone **[L]** Inter-professional **[M]** Entail advocacy and safe guarding	Fatally flawed paper
Focht-New, 2012, USA. **(S29)**	Describe RNs experiences of teaching for people with IDD	*N* = 23 RNs Gender not known Learning disability community trust	Grounded theory	Interviews, focus group interviews, non-participant observations Constant comparison	Translatable 2nd order concepts *n* = 1 Non-translatable 2nd order concepts *n* = 1	**[B]** Rest its foundation on trust **[G**^a^**]** Raise the bar in care for this patient group **[H]** Acknowledge the person behind the label of disability	Fatally flawed paper
Hellzen et al., 2004, Sweden. **(S5)**	Illuminate the meaning of being a nurse for an extremely provoking patient with ID	N = 8 Enrolled nurses ♀ 5 + ♂ 3 Group Dwelling	Phenomenology	Narrative interviews Constant comparison	Translatable 2nd order concepts *n* = 2 Non-translatable 2nd order concepts *n* = 1	**[J**^a^**]** Take unpredictable situations into account	Satisfactory paper
Lee & Kiemle, 2014, United Kingdom. **(S7)**	Gaining an in-depth understanding of the day-to-day experience of nurses working with people diagnosed with both PD and ID	N = 9 RNs ♀ 7 + ♂ 2 Medium-secure and low secure units	Interpretive phenomenology	Semi-structured interviews, Interpretative phenomenological analysis	Translatable 2nd order concepts *n* = 3 Non-translatable 2nd order concepts = 0	**[A**^a^**]** Based on long-term relationships **[F**^a^**]** Work against negative attitudes and alienation **[H]** Acknowledge the person behind the label of disability **[J**^a^**]** Take unpredictable situations into account	Satisfactory paper
Li & Ng, 2008, United Kingdom. **(S8)**	Explore nurses´ experiences in caring for dying patients with profound learning disabilities	*N* = 5 RNs ♀ 3 + ♂ 2 Residential homes in one PCT in the South of England	Generic qualitative design	Open-ended semi-structured interviews Constant comparison	Translatable 2nd order concepts *n* = 2 Non-translatable 2nd order concepts = 0	**[A**^a^**]** Based on long-term relationships **[K]** Knowledge and skills beyond the diagnosis (here IDD) **[L]** Inter-professional	Fatally flawed paper
Marsham,2011, United Kingdom. **(S31)**	Explore the experiences of RNIDs communicating with adults with ID who use	N = 8 RNLDs ♀ 8	Descriptive phenomenology	Semi-structured interviews Phenomenological analysis	Translatable 2nd order concepts *n* = **6** Non-translatable 2nd order concepts = 0	**[C**^a^**]** Go beyond verbal communication alone **[E]** Include relevant others to offer quality care [G*] Raise the bar in care for this patient group **[H]** Acknowledge the person behind the label of disability **[L]** Inter-professional **[N**^a^**]** Understand the complexity of this of patient group	Fatally flawed paper
Martin et al., 2012a, Ireland. **(S10)**	Explore the experiences of RNIDs communicating with adults with ID who use non-verbal communication	N = 8 RNLDs ♀ 8 Irish residential service for people with IDD	Phenomenology	Semi-structured interviews Phenomenological analysis	Translatable 2nd order concepts *n* = 1 Non-translatable 2nd order concepts = 0	**[A**^a^**]** Based on long-term relationships **[B]** Rest its foundation on trust	Satisfactory paper
Martin et al., 2012b, Ireland. **(S11)**	Explore the experiences of RNIDs communicating with adults with ID who use non-verbal communication	N = 8 RNLDs ♀ 8 Irish residential service for people with IDD	Phenomenology	Semi-structured interviews Phenomenological analysis	Translatable 2nd order concepts *n* = 2 Non-translatable 2nd order concepts *n* = 1	**[C**^a^**]** Go beyond verbal communication alone **[L]** Inter-professional	Fatally flawed Paper
Morton-Nance & Schafer, 2012, United Kingdom. **(S12)**	Explore the experiences of RNLD and district nurses caring for people with a ID at the end of their lives	N = 6, 3 RNs & 3 RNIDs ♀ 6 Two specialist health care settings	Descriptive phenomenology	Semi-structured interviews Thematic analysis	Translatable 2nd order concepts *n* = 5 Non-translatable 2nd order concepts = 0	**[A**^a^**]** Based on long-term relationships **[C**^a^**]** Go beyond verbal communication alone **[D]** Forward planning **[E]** Include relevant others to offer quality care **[F**^a^**]** Work against negative attitudes and alienation **[G**^a^**]** Raise the bar in care for this patient group **[H]** Needing acknowledge the person behind the label of disability **[L]** Inter-professional	Fatally flawed paper
Ndengeyingoma & Ruel, 2016, Canada. **(S21)**	Explore nurses´ representations of caring for people with ID, intervention strategies they current use, and to identify needs to ensure quality care	*N* = 18 RNs ♀ 14 + ♂ 4 2 general hospitals, 1 mental health hospital and 7 community care centres	Generic qualitative design	Semi-structured interviews Thematic analysis	Translatable 2nd order concepts *n* = 3 Non-translatable 2nd order concepts = 0	**[C**^a^**]** Go beyond verbal communication alone **[E]** Include relevant others to offer quality care **[G**^a^**]** Raise the bar in care for this patient group **[K]** Knowledge and skills beyond the diagnosis (here IDD) **[L]** Inter-professional **[N**^a^**]** Understand the complexity of this patient group	Satisfactory paper
Slevin & Sines, 2005, United Kingdom. **(S26)**	Investigate the roles of community nurse for people with ID when caring for clients, and their careers, when the client is a person who indulges in challenging behaviours	*N* = 22 RNLDs Gender not known1 UK NHS trusts	Grounded theory	In-depth face-to-face interviews Constant comparison	Translatable 2nd order concepts *n* = 6 Non-translatable 2nd order concepts *n* = 3	**[A**^a^**]** Based on long-term relationships **[D]** Be forward planning **[E]** Include relevant others to offer quality care **[F**^a^**]** Work against negative attitudes and alienation [G*] Raise the bar in care for this patient group **[I]** Based on evidence-based practice **[L]** Inter-professional **[N**^a^**]** Understand the complexity of this patient group	Satisfactory paper
Sowney & Barr, 2006, Northern Ireland. **(S13)**	Explore the experiences of nurses working in accident and emergency departments in the assessment and provision of care to adults with ID	*N* = 27 RNs Gender not known Hospital, accident and emergency departments	Generic qualitative design	Focus group interviews Thematic analysis	Translatable 2nd order concepts *n* = 1 Non-translatable 2nd order concepts *n* = 1	**[E]** Include relevant others to offer quality care **[K]** Knowledge and skills beyond the diagnosis (here IDD)	Key paper
Taua & Farrow, 2009, New Zealand. **(S14)**	Identify and describe current nursing practice within an inpatient ID service and to identify factors that influence current nursing practice within an inpatient ID service	N = 5 RNs Gender not known Inpatient ID service	Ethnography	Observations and semi-structured interviews Ethno semantic analysis	Translatable 2nd order concepts *n* = 1 Non-translatable 2nd order concepts = 2	**[J**^a^**]** Take unpredictable situations into account	Key paper

^a^ = LOA synthesis interpreted to represent a tentatively distinct conceptualization of nursing

### Line of argument synthesis

Our LOA synthesis represents 18 studies contributing 47 second-order concepts we assessed as translatable. We categorised ten (55%) of these as FFPs; these contributed 28 translatable second-order concepts (59.5%) to the synthesis. We categorised six studies as SPs which contributed 17 translatable second-order concepts (36%). We categorised two papers as KPs that contributed two translatable second-order concepts (4%). Only second-order concepts assessed by two independent reviewers as translatable were included in the synthesis. We assessed 14 second-order concepts as non-translatable, representing eight studies which were, therefore, excluded from the synthesis (Table 3).

We interpreted our synthesis to represent 14 LOA syntheses (coded from A to N), helping us to conceptually clarify the experience of nursing patients with IDD. The LOA synthesis, ‘nursing experienced as needing to *take unpredictable situations into account*’ [41–44] was only represented by studies assessed as SPs or KPs. The LOA synthesis ‘nursing experienced as needing to *entail advocacy and safe guarding*’ [45, 46] was only represented in studies assessed as FFPs. The remaining 11 LOA syntheses were represented equally among the studies, regardless of their quality assessment.

#### Based on long-term relationships [A]

The nursing of patients with IDD rests on having a long-term bond between nurse and patient; this stood out as a recurring experience. Such a relationship was experienced as a prerequisite to be able to understand patients with IDD [42, 45, 47–52] and as a prerequisite when there was a need to add a therapeutic component to nursing [51] or when nursing aimed to empower patients [52]. In some cases [42], this LOA synthesis also reflected the idea that it could be challenging to forge the long-term relationships needed for nursing this group of patients. In other cases [52], the RNs felt they needed to first create a relationship with relevant others before being able to create a long-term relationship with the patient.

#### Rest its foundation on trust [B]

Gaining the trust of patients with IDD stood out as relevant [48, 50, 53]. This could be an experience of nursing in general but is particularly relevant in order to offer patients with IDD a secure, stable and predictable situation while making sure they are understood [50]. The LOA synthesis could also reflect an experience of nursing needing to ensure patients cooperated in different care activities, such as tests and examinations [48]. Basing nursing on trust could at times be experienced as needing to create a trusting alliance with relevant others before being able to create the same alliance with patients [53].

#### Go beyond verbal communication alone [C]

The need to be able to communicate nonverbally was a recurring experience of nursing patients with IDD [45, 48, 51, 54–56]. The RNs experienced nonverbal communication as a complex skill they needed to use constantly when interacting with this group of patients [48, 55]. In some cases, this experience was closely related to achieving nursing goals, such as ensuring no care needs go unmet and to be able to deliver safe, optimal care for patients [45, 48, 51, 54, 55].

#### Be forward planning [D]

Nursing for this group was experienced as needing to have a long-term perspective, that is, making it the norm to be one step ahead in terms of taking preventative measures [52], particularly for older patients with IDD [49], to constantly plan ahead and to double check everything [51]. This was particularly reflected as an experience of nursing when the care delivered needed to be able to offer the patients a relevant environment as they grow older and offer support in retirement, loss and bereavement [48, 49].

#### Include relevant others to offer quality care [E]

Dependence on and the engagement of custodians and relevant others was another experience of nursing this patient group [49, 51, 52, 54, 56, 57]. In the LOA synthesis, this experience was often reflected in care that aimed to offer patients with IDD relevant support [49, 51, 52, 54, 57] or care aiming to secure and improve specific high-quality services [51] and secure safe health services [56].

#### Work against negative attitudes and alienation [F]

The synthesis reflected that the RNs could experience negative attitudes towards this group of patients. The need to actively engage in working against negative attitudes was therefore a distinct experience of nursing vis-à-vis this group of patients [42, 46, 49, 51, 52, 58]. In some cases, this experience of nursing was related to counteracting unequal and poorly delivered health care services [46, 49, 51, 52]. The LOA synthesis also reflected that the RNs at times had negative attitudes towards this patient group and could be influenced by their own perceptions of behavioural gender links [42].

#### Raise the bar in nursing for this patient group [G]

This LOA synthesis reflected a repeat experience of nursing needing to actively engage in educating, informing and teaching colleagues and relevant others about the care and needs of the patients [49, 51–54, 56, 58]. In some cases, the experience of nursing and the general need to raise the level of competence and knowledge was related to achieving the goals of nursing on both an organisational and an individual level. The goals could be to offer patients relevant support [56], personalised and safe health care services [54], secure and improve high-quality palliative care services [51], reduce patients’ challenging behaviour [52] and work towards societal approval and inclusion and against unequal and poorly delivered health care services [53]. In other cases, this experience of nursing reflected minimal exposure to this patient group during nursing education [49, 53], thus reflecting a need for further specialisation to be able to act as a source of advice and support for other RNs [56, 58]. The latter experience was also interpreted as affecting nurses’ confidence and competence in caring for this patient group [56].

#### Acknowledge the person behind the label of disability [H]

Acknowledging the actual person behind the label of IDD stood out as an important part of the experience of nursing. This was experienced as a necessity for tailoring nursing to this patient group by, for example, incorporating a person-focused approach [49, 51]. In particular, this could mean that nursing focused on abilities instead of disabilities [56], especially where the latter was experienced as minimising the influence of the negative label of IDD [42]. Their experience of nursing could also be reflected in educational activities where RNs could feel a strong need to raise awareness of this patient groups’ often low self-worth and disadvantages [53].

#### Evidence-based practice [I]

Nursing for this group of patients could be experienced as needing to be based on best practices and evidence-based practices (EBPs) [49, 52]. This was particularly reflected in nursing care plans focusing on behavioural approaches to enable RNs to deal with CB among patients with IDD [52], but also in general because nursing could be experienced as needing to be based on experience, education and research [49].

#### Take unpredictable situations into account [J]

Nursing patients with IDD, particularly patients displaying behaviour that challenged, could be experienced as testing, unpredictable and dangerous [41–44]. The RNs experienced a need to be on the alert and prepared for volatile, unpredictable situations [43, 44] and to be prepared to control chaos and turmoil [42]. In some cases, this experience of nursing was related to being able to deliver safe care in all types of environments, whether high risk or low risk [43, 44]. Nursing could also mean experiencing destructive thoughts about patients and a variety of different negative emotions about the patients [41–43]. In some cases, this meant experiencing a need to emotionally distance oneself and ‘close off’ [42]. This LOA synthesis also reflected experiences of a need to focus on personal protection and safety [43] and fears that violence and abnormality could become the norm when nursing this group of patients [44].

#### Knowledge and skills beyond the diagnosis (here IDD) [K]

Nursing for patients with IDD could mean experiencing a lack of knowledge and skills relating to IDD [54, 57]. Nursing could also be experienced as extra challenging when physical ill health became part of the equation [47] or when not knowing where to go for support when patients displayed signs of physical ill health [57]. Lacking knowledge about physical ill health in general could also result in an experience of ill health being under- or over-diagnosed [57] or that nursing did not meet the patient’s actual needs [54].

#### Inter-professional collaboration [L]

Nursing that actively engaged in inter-professional collaborations with significant others around the patient stood out as an evident experience of nursing patients with IDD. Working inter-professionally was experienced as an important tool for ensuring safe care [45, 47, 51, 52, 55] but also for being able to offer structured and organised care [56] and nursing where there were no unmet care needs [54].

#### Entails advocacy and safe guarding [M]

Nursing patients with IDD could be experienced as needing to take on the role of guardian and spokesperson, that is, speaking up and acting on a patient’s behalf [45, 46]. This LOA synthesis reflected experiences of nursing entailing a supporting role in allowing the patient with IDD to be in control of his/her life and autonomy [46] and in ensuring all needs were met despite at times experiencing conflicting wishes between the RNs and the patients [45].

#### Understand the complexity of this patient group [N]

This LOA synthesis reflected that nursing patients with IDD could be experienced as more complex than nursing other patients. The need to actively let the patients set the pace for care, surveillance and/or assessment of needs, that is, ‘to allow it to take time’, was experienced as a major part of the complexity. Allowing time was viewed as vital to achieve well-executed and optimal nursing for this group of patients [52, 54, 56, 58]. The complexity became particularly visible when care organisations did not support patients’ need for long-term nursing care [58] or their individual resource allocation needs [54]. In particular, the latter was experienced as restricting RNs’ abilities to deliver optimal care for this patient group [54].

## Discussion

The RNs’ experiences of nursing patients with IDD were interpreted to be reflected in 14 individual LOA syntheses. We propose that six of these LOAs (Table 4) are tentatively distinctive and a unique conceptualisation of RNs’ experience of nursing regarding this group of patients regardless of context. We suggest that the remaining eight LOAs represent conceptualisations of nursing already collectively described and known independent of patient group and contexts. Our discussion begins by addressing the six tentatively unique conceptualisations and ends by briefly addressing the remaining eight general conceptualisations of nursing.

**Table 4:** Conceptual understanding of Nursing for patients with IDD

LOA Synthesis	Nursing for People with IDD*	LOA Synthesis	Nursing Per Se
A	Based on long-term relationships	B	Rests its foundation on trust
C	Go beyond verbal communication alone	D	Be forward planning
F	Work against negative attitudes and alienation	E	Include relevant others to offer quality care
G	Raise the bar in nursing for this patient group	H	Acknowledge the person behind the label of disability
J	Take unpredictable situations into account	I	Based on evidence-based practice
N	Understand the complexity of this patient group	K	Knowledge and skills beyond the diagnosis (here IDD)
		L	Inter-professional collaboration
		M	Entails advocacy and safe guarding

* = LOA synthesis interpreted to represent a tentatively distinct conceptualization of nursing

Without the presence of a long-term relationship in care, nursing patients with IDD is likely to be experienced as a challenge and as being at risk of failing to deliver quality care. In the LOA synthesis ‘nursing experienced as needing to be *based on long-term relationships*’, it was reflected that reading, understanding, supporting and empowering patients with IDD was not likely to take place without nursing that includes strategies such as building long-term relationships with patients [42, 45, 47–52]. Regardless of patient group or context, it is well known that one of the most reported essential qualities of nursing is the relationship between an RN and a patient [59–61]. In her literature review including studies outside our context, Shattell [62] suggests that in normal cases and under favourable circumstances nurse–patient relationships occur and work in short-term care interactions. However, we propose that in the majority of health care contexts such a relationship is not aligned to a long-term perspective and is particularly needed when nursing this group of patients, since creating a foundation for trust in care becomes a prerequisite for nursing. Belcher and Jones [63] and others [64] have identified trust as a crucial component in establishing an effective nurse–patient relationship. They ascertain that the RN is the key to developing trust, as the patient is in the RN’s environment and is therefore in a vulnerable position [64]. Patients with IDD can be said to be in an extraordinarily vulnerable position. Not knowing the patient has been found to result in RNs not being sufficiently confident to build a relationship, resulting in inadequate patient care [63]. The nurse–patient relationship has also been described as an important factor for patient participation in care [63, 65]. It is therefore reasonable to suggest that nursing for this patient group not based on a long-term relationship can adversely influence RNs’ ability to interpret patients’ care needs, which could result in lack of reliance, unmet needs and unsafe care. According to Gámez [66], the nurse–patient relationship should concentrate on the needs, limitations and potentials of the individual person. It is noteworthy that we could not identify any published studies researching the nurse–patient relationship in the context of this patient group. The majority of studies identified implied that the topic has not been focused on since early in the twenty-first century, suggesting this is an area of nursing in need of more in-depth knowledge development and focus.

Communication in nursing patients with IDD clearly demands skills and competences in communication strategies above and beyond the spoken word. The LOA synthesis ‘nursing experienced as needing to *go beyond verbal communication alone*’ reflected how not being able to communicate impacts the assessment of care needs and the quality of care provided and results in insufficient and unsafe care [45, 48, 51, 54–56]. Our findings are supported by Fisher [67], who states that patients with IDD often require information in various formats to help them understand and communicate. Successful communication demands cognitive efforts (ibid) and both verbal and nonverbal cues [68]. Even if patients with IDD are able to communicate verbally, difficulties in understanding the spoken word can occur [67]. Such difficulties can be exacerbated by health care professionals being prone to using jargon [69], especially as patients with IDD often interpret words and sentences literally [70]. Furthermore, it is not uncommon for RNs and other health care professionals to have problems understanding what IDD patients are trying to convey, thus resulting in difficulty assessing their needs [70]. Hemsley and colleagues [71, 72] state that nonverbal communication must be used and properly understood to fully achieve the goals of communication. Otherwise the patient’s mannerisms and behaviour can be misunderstood and the patient seen as non-compliant when in fact the problem lies in the health care professional’s failure to understand the patient’s usual methods of communicating [71, 72] Therefore, there is support for the supposition that challenges in interpreting communication cues result in difficulties assessing care needs [70] and in inadequate and unsafe care [72]. It could be argued that RNs should encourage patients with IDD to bring a friend, a relative or a custodian as this could increase RNs’ ability to understand the patient and hence being able to offer relevant care.

It is noteworthy that nursing patients with IDD needs to include strategies aimed at shattering the stigmatisation that still appears to be associated with this group of patients and that became apparent in the LOA synthesis ‘nursing experienced as needing to *work against negative attitudes and alienation*’. Negative attitudes towards patients with IDD was found to derive from both the surrounding society and the care context [42, 46, 49, 51, 58], as well as from RNs themselves [42]. Our findings imply that this could lead to unequal and poorly delivered health care services for this patient group [46, 49, 51]. In a study by Lewis and Stenfert-Kroeses, RNs were found to hold significantly less positive attitudes when nursing patients with IDD compared to when nursing patients with a physical disability [15]. It was common that this patient group would be experienced by the RNs as easily distressed, aggressive and less co-operative. Additionally, it was less likely that RNs included the patients in decisions about their care, as they stated they would, for example, not explaining treatment plans or assessing if a patient was in pain [15]. In a study by Cumella and Martin [73], RNs described feeling awkward and embarrassed when interacting with this patient group. Others report equally distressing findings, such as relevant others hearing negative comments by health care staff about their relative with IDD [74] and patients with IDD experiencing health care staff being judgemental regarding their capabilities, something that obviously was found to influence their willingness to seek health care [75]. Bradbury and colleagues [12] state that discrimination, anxiety and fear are often underpinned by lack of knowledge. This state of affairs is distressing in the context of nursing. One plausible explanation for this unacceptable situation might be that in the majority of European countries nursing education still does not offer advanced courses and/or programs dealing with this nursing speciality, in contrast to the United Kingdom and Ireland. Bachelors nursing students are seldom given any opportunity to interact with this patient group during clinical placements, and neither do most course curricula include any substantial learning objectives regarding the knowledge needed to nurse this patient group. Therefore, higher educational institutions (HEIs) offering nursing education need to develop courses and programs that equip students with the knowledge and skills needed to provide this patient group with the same quality health care as provided to the rest of the population.

Without the right knowledge of IDD and how to care for these patients, nursing is likely to be experienced as a challenge. Challenges on both an individual and an organisational level, such as failing to deliver quality, safe and supportive care, became evident in the LOA synthesis ‘nursing experienced as needing to *raise the bar in nursing for this patient group*’ [49, 51–54, 56, 58]. We know that patients with IDD can experience difficulties when interacting with their surroundings and are therefore a vulnerable population prone to placing great demands on the health care system. In addition, the research indicates that RNs do not feel adequately prepared [76] or knowledgeable enough to support these patients or to meet their needs [15]. This can cause major obstacles for RNs in providing appropriate and adequate health care [67]. It is not uncommon that nursing is dependent on support people, such as custodians and/or relevant others, to be able to care for this patient group [76]. Northway and colleagues [2] state that this raises questions about social justice and equal rights, as patients with disabilities have the same right to the highest attainable standard of health care as everyone else. The research has highlighted that RNs educated about learning disabilities are better equipped to provide good quality care to this group. In those European countries offering this type of education and service, RNs with knowledge of learning disabilities, aka IDD, have been found to play a crucial role in coordinating care between the community and the hospital and in increasing knowledge, awareness and understanding of this group of patients among health care staff [74, 77]. Therefore, exposure to and enhancing knowledge about this patient group must become an educational, professional and nursing research priority.

The ability to manage the unpredictable interactions due to CB and the risks CB can pose for fellow patients and health care staff is essential in nursing this group of patients. The LOA synthesis ‘nursing experienced as needing to *take unpredictable situations into account*’ reflected how RNs can experience a constant need to be vigilant and prepared for unpredictable situations in which they need to take control quickly to ensure safe care [41–44]. The above is well established, as CB has been associated with IDD since the mid 1990s. According to Farrell et al. [78], one of the most commonly quoted definitions in IDD literature is Emerson et al.’s [79] description of CB ‘as behaviour of such intensity, frequency or duration that the physical safety of the client or others is at risk, or as behaviour which is likely to limit or deny access to normal facilities’ [78]. This definition accurately reflects the experiences of RNs in our review. Our synthesis also reflected how unpredictable situations could lead to RNs experiencing moral distress (i.e. anger and negative thoughts about patients), resulting in guilt and a need to distance themselves from the situation and the patient. This is corroborated by, for example, Whittington and Burns [80]. They found that CB caused a dilemma for health care staff: should CB be viewed as communicating needs or as a behavioural problem? Staff also discussed how to deal with the unpleasant feelings CB can evoke; unfortunately, their main strategies were to avoid the patient and/or to shut themselves off emotionally [80]. Farrell et al. [78] state it is not uncommon for health care staff to report negative feelings, such as guilt, fear and powerlessness, in relation to CB. The implications of this for nursing patients with IDD, particularly in regard to quality of care, are problematic. Training and educational interventions are therefore required to support RNs in nursing these patients in a clinical context. We suggest that educational models, such as the S_VES_OS [78] with its three core domains of self, others and setting, as the basis for developing educational objectives could be the foundation of such educational clinical interventions.

Delivering ‘health care and nursing as usual’ is likely to fail in terms of meeting the needs of patients with IDD. This became obvious in the LOA synthesis ‘nursing experienced as needing to *understand the complexity of this patient group*’ [52, 54, 56, 58]. The experiences reflected that a major part of the complexity was a result of an organisation not supporting patient needs for long-term nursing, more time, a slower pace and other resources that differ compared to those required by general patients. It therefore seems reasonable to conclude that current health care systems might not be the best for this group of patients with complex needs as they do not seem to take into account how their cognitive functions differ from people in the general population. Therefore, these systems need to adapt their services. There is evidence of inadequate attention to care needs and inadequate access to health care, including oral care [81, 82]. Others have found that the length of appointments for patients with IDD is the same as for other patients [11, 83] and that RNs’ experiences reflect a lack of time when nursing patients with complex needs [84]. This is noteworthy, especially as we know that this patient group has a higher likelihood of adverse health outcome [81] and consequently are more prone to needing health care. Implementing careful individual planning and care coordination by case managers would be a way for health care systems to acknowledge and deal with the complexities associated with this group of patients.

### Nursing per se

Eight LOA syntheses (Table 4) were interpreted to tentatively represent a conceptualisation of nursing per se. i.e. a conceptualisation of nursing that has been interpreted elsewhere and describes a universal experience of nursing in general. We will, therefore, only discuss them very briefly.

Trust is a crucial component of nursing despite the caring context or if nursing needs to be based on a short- or long termed relationship. Therefore, not surprisingly, nursing was also experienced here as needing to ‘*rest its foundation on trust*’ [48, 50, 53]. Our LOA synthesis reflected that if trust was not established in nursing, the patients were unlikely to cooperate in their care. Belcher and Jones [63] support this finding, having found that if trust is not established between a RN and a patient it could result in an unconfident RN and an uncooperative patient. Others [85] have shown that patient satisfaction with care is positively related to their trust in the RN. In addition to a trusting relationship, all nursing must involve a broad and long-term perspective, as supported by the LOA synthesis ‘nursing experienced as needing to *be forward planning*’ [49, 51, 52]. It reflected that not being one step ahead makes it difficult to adequately support patients in terms of their health. This is of importance regardless of context, as advanced care planning has been found to reduce unnecessary transfers to hospital and complications associated with being in hospital [86].

In order for nursing to ensure that essential human needs are meet i.e. the fundamentals of care [87], and regardless of what context nursing occurs in, RNs might at times experience nursing as needing to ‘*include relevant others to offer quality care*’ [49, 51, 52, 54, 56, 57]. Our LOA synthesis reflected that in nursing, the RNs aiming to offer support or deliver appropriate health care services had to depend on the engagement of relevant others. Research has revealed that the involvement of relevant others and/or custodians is a crucial component in delivering care, particularly for vulnerable patient groups [88, 89]. However, delivering fundamentals of care e.g. feeding, drinking, washing and changing is a mandatory practice competence within the remit of the RNs role in care (i.e. the duty of care), that should not be left to significant others to assure that the essential needs of relatives with IDD are properly meet when in hospital, as evident in the report from Mencap [16] ‘*Death by indifference: 74 deaths and counting’*. This is particularly relevant since a reasonable inclusion of relevant others in nursing care fits well with the core competency of person-centred care (PCC) that our next LOA stresses, i.e. acknowledging that all patients are people. This became evident in the LOA ‘nursing experienced as needing to ‘*acknowledge the person behind the label of disability*’ [42, 49, 51, 56]. The RNs’ experiences reflected that to be able to offer relevant nursing, their focus needed to be on the patients’ abilities and not on their disabilities. This is translatable regardless of whether a patient’s disability is cognitive or physical. Such findings are encouraging and promote the recent focus [90] on PCC [91], which might have a sustainable future in nursing and health care. Others support this; for example, Andersson and colleagues [92] found that the starting point for nursing was when the RN acknowledged and saw the person behind the patient.

Evidence based practice (EBP) is a necessity for all health care, as indicated by one of the conceptualisations in our study, ‘nursing experienced as needing to be based on *evidence-based practice*’ [49, 52]. The RNs found that in order to care for a patient based on specific aims, such as reducing CB, and to provide good care in general, nursing needed to be grounded in EBP. The latter is described as a problem-solving approach to the delivery of health care that integrates the best evidence from studies and patient care data with clinicians’ expertise and patient preferences and values [91]. EBP has been shown to result in higher quality care, improved patient outcomes, reduced costs and greater RN satisfaction in comparison to care not grounded in evidence-based knowledge [93]. Relevant quality nursing for patients can only be a reality if RNs possess a broad knowledge base and employ nursing practices based on evidence. This became evident in the LOA synthesis ‘nursing experienced as needing *knowledge and skills beyond the diagnosis*’ [47, 54, 57]. Our synthesis reflected experiences coloured by insufficient competences, both when IDD and somatic health were concerned. It is fair to acknowledge that this is not unique for this group of patients. Research has highlighted similar experiences for general RNs in relation to mental health [94] and for mental health RNs in relation to physical health [95]. In a recent Cochrane review [96] assessing the effects of organisational interventions on mental and physical health care services for people with IDD, the authors concluded there is very limited evidence on the organisation of health care services for people with IDD. Therefore, there is a pressing need for high-quality health services research to identify optimal health services for patients with IDD and concurrent somatic problems.

With the increasing complexity of care, working in inter-professional teams is important; therefore, nursing was experienced as needing to be ‘*inter-professional*’ [45, 47, 51, 52, 54–56]. Our LOA synthesis reflected that working in inter-professional teams was an important tool in meeting care needs and assuring safe, quality care. Working in interprofessional teams is known to result in improved quality and decreased health care costs [97]. In a study by Andersson et al. [92], RNs perceived that as part of an interprofessional team they were transferring important information and knowledge about the patients. Teamwork is also known to improve patient planning, is clinically more efficient and supports PCC [98] and is therefore a vital part of modern health care services in general. Standing by patients and speaking up regarding their best interests has always been an important part of nursing, particularly as nurses are part of an interprofessional team. Not surprisingly, nursing was also experienced as needing to ‘*entail advocacy and safe guarding*’ [45, 46]. Our synthesis reflected that a patient would most likely lose his/her autonomy if the RN did not stand up and speak for that patient; otherwise, the result is unmet and missed care needs. The act of advocacy is of great importance and entails RNs being attentive, present and prepared to support patients in all situations whilst at the same time giving patients freedom to decide the extent to which they want to participate [92, 99].

### Strengths and weaknesses of this study

The relevance of our findings needs to be critically assessed by the reader based on our choice and execution of method. The indexing and archiving of qualitative research has advanced since Noblit and Hares’ [27] seminal meta-ethnography, but it is well known [35] that identifying relevant qualitative research through developing search strings resulting in high sensitivity and specificity, is still a challenge. Our search strategy was therefore developed and systematically trialled in close collaboration with specialist librarians. Free-text terms and database-specific subject headings, manual searches of reference lists and checking of published systematic reviews were used to identify relevant studies. We acknowledge that despite this, some qualitative studies might have been missed.

Our inclusion criteria were designed to identify the most relevant qualitative studies for our review question and conceptually rich enough that they contained concepts for translation to support the meta-ethnographic approach. To avoid omitting research of potential value for our synthesis, both generic qualitative studies and qualitative studies guided by an explicit set of philosophical assumptions in the form of one of the known qualitative methodologies [100] were included. Synthesising studies with different philosophies and methodologies might lead to bias in terms of the range and nature of qualitative research synthesised. To reduce this risk, we controlled for the possible influences of study design throughout our processes. Of note, a majority of the LOA syntheses were found to be underpinned by second-order constructs from studies conducted using one of the established classical designs [100], regardless of how the study had been quality assessed. We suggest that one of the strengths of the meta-ethnographic approach is the combination of findings from multiple sources. According to Jamal and colleagues [101], this increases legitimacy and supports a move beyond narrative accounts to the development of higher-order explanations of the phenomenon in *foci*.

There is currently a discussion about i) the importance of quality appraisal per se and ii) whether eligible studies assessed as methodologically weak should remain in the synthesis [39, 102]. This inspired us to use Toye and colleagues’ [29] approach to appraise both study designs and concepts. Our team appraised the quality of the study’s concepts (i.e. second-order concepts) as translatable or non-translatable and the methods used to categorise studies as FFPs, SPs or KPs [29]. Our experience of including all translatable concepts despite the categorisation of included papers corroborates their methodological approach. We found that KPs or FFPs were not mutually exclusive, as both could represent individual translatable second-order concepts as conceptually rich and insightful.

By comparing translations, meta-ethnography aims to provide a deeper conceptual understanding of the phenomenon under investigation [27]. Therefore, we took great care to ensure our second-order concepts were firmly grounded in the primary studies by moving back and forth between the studies’ idiomatic translations and the LOA synthesis, thus striving to be true to the meaning of the original author. We kept thorough notes during data extraction in order not to lose the conceptualisation. As the majority of our main LOAs were fairly well represented throughout the 18 included studies regardless of their quality assessment, we suggest that our synthesis reflects well how both general and specialist RNs experiences of nursing patients with IDD can be generalised across a European Health Care context.

## Conclusions

The relevance of this meta-synthesis lies in the implications the RNs’ experience has for nursing. Our findings suggest that negative stigmatisation, attitudes and alienation still seem to surround this group of patients. Furthermore, the health care needs of patients with IDD might not yet be adequately addressed despite the fact that they have a right to the same highest attainable standard of health care as others. This indicates that RNs might be compromised in their ability to contribute to reducing health disparities for these patients.

Our findings also suggest that services were not designed to meet the health  care needs of patients with IDD. Complex health conditions, communication difficulties and challenging behaviours were some of the challenges the RNs experienced while nursing this patient group. Careful individual planning, care coordination and case management could be ways for the system to acknowledge the complexity of this group of patients. Additionally, to ensure that the fundamentals of nursing are met at all times, RNs need to plan and deliver nursing in a structured and systematic way. For example, our findings indicate that creating a long-term relationship with the patient with IDD and going beyond verbal communication alone could be one possible way to enable RNs to work in a person-centred way. Particularly as our findings also implied that focusing on the person behind the disability label, i.e. PCC in encounters between patients with IDD and RNs could be one way to secure quality nursing for patients with IDD. This seems reasonable to suggest as such an approach could support the forging of much needed long-term nurse–patient relationships, which in turn would likely enhance RNs’ ability to understand patients, regardless of their method of communication. Nursing care models focusing on PCC might be adequate strategies in supporting the configuration of mainstream health care contexts to meet the health needs of patients with IDD. Large scale experimental studies testing the effectiveness of PCC nursing care models to this group of patients in clinical practice are therefore warranted.

Our review also suggests that with some few exceptions, relatively little attention has so far been given to patients with intellectual disability disorders within general nursing and in nursing research. For general nursing, it is important to consider that nowadays this rather large group of people no longer reside in large institutions and that many of them live longer compared to previous generations. Hence it is almost certain that general RNs, often in the frontline of home and primary care, will be of great importance for the health and care for this patient group. For nursing research, focus seems to be on challenging behaviour, mental health problems and communication problems. However, it is vital to not forget that this is not a homogenous patient group. Consequently, there is more to explore and knowledge to gain within nursing and health care than around these few negative IDD phenomena. It therefore, seems reasonable that RNs in countries (e.g. UK and Ireland) with a specialist education take the lead in building up a relevant body of knowledge in some of those areas of importance reflected here. If not addressed, the vital evidence needed for nursing practice will be lacking, the essence of nursing care not captured and its contribution not accurately valued.

A reoccurring theme in our systematic literature review was the lack of awareness and of relevant knowledge experienced by the RNs, very likely being the main reason for the ‘otherness’ that still surrounds patients with IDD. The only reasonable remedy for this is education. The main question is how to deliver this, as not all European countries, with the exception of United Kingdom and Ireland, offer relevant under- and post graduate programme or courses focusing on patients with IDD within different health science curricula. Specialist education for nurses caring for patients with IDD must be put on the agenda in all European countries so that the contribution that RNs on the front line of care can make in reducing any possible health disparities in this group of patients not are compromised.

## Additional files

Additional file 1: Prisma Checklist. (DOC 63 kb)

Additional file 2: Search strings. (DOCX 28 kb)

Additional file 3: Studies excluded after full text reading. (DOCX 22 kb)

## Abbreviations

CB: Challenging Behaviour
EBP: Evidence-Based Practice
ENTREQ: Enhancing transparency in reporting the synthesis of qualitative research
HEI: Higher educational institutes
ICD: International Classification of Diseases
IDD: Intellectual disability disorder
LOAs: Line of argument synthesis
PCC: Person Centred Care
RN: Registered Nurse
S_VES_OS: Self, values, emotional reactions, skill repertoire, Other, Setting
