# Supplementary material for: Nurses’ experiences of caring for patients with intellectual developmental disorders: a systematic review using a meta-ethnographic approach
Source: BMC Nurs. 2018 Dec 3;17:51. doi: 10.1186/s12912-018-0316-9 (PMC6276187; doi:10.1186/s12912-018-0316-9)
Supplement: Supplementary file 2 — Search strings. (DOCX 28 kb) [file 12912_2018_316_MOESM2_ESM.docx]

**Additional file 2.** Search blocks

| **CINAHL Search block 1:** Intellectual developmental disorder [2017-11-16] **No items found** | |
| --- | --- |
| 1. asperger syndrome [MeSH] | 1619 |
| 1. autism spectrum disorder [MeSH] | 1526 |
| 1. autistic disorder [MeSH] | 17 711 |
| 1. down syndrome [MeSH] | 22 086 |
| 1. intellectual disability [MeSH] | 86 516 |
| 1. learning disorders [MeSH] | 20 136 |
| 1. asperger* [title/abstract] | 1963 |
| 1. autism* [title/abstract] | 29 213 |
| 1. autistic dis* [title/abstract] | 1526 |
| 1. down syn* [title/abstract] | 11 935 |
| 1. intellectual developmental dis* [title/abstract] | 80 |
| 1. intellectual dis* [title/abstract] | 10775 |
| 1. learning dis* [title/abstract] | 9158 |
| 1. mental retard* [title/abstract] | 26 043 |
| 1. pervasive developmental dis* [title/abstract] | 1886 |
| 1. *1 OR 2 OR 3 OR 4 OR 5 OR 6 OR 7 OR 8 OR 9 OR 10 OR 11 OR 12 OR 13 OR 14 OR 15* | *155330* |

| **CINAHL Search block 2:** Nursing [2017-11-16] **No items found** | |
| --- | --- |
| 1. health occupations [MeSH] | 1 514 851 |
| 1. health personnel [MeSH] | 426 117 |
| 1. licensed practical nurses [MeSH] | 31 |
| 1. nurses [MeSH] | 77 365 |
| 1. patient care team [MeSH] | 59 554 |
| 1. ”allied health care personnel” [title/abstract] | 5 |
| 1. ”allied health care staff” [title/abstract] | 0 |
| 1. allied health care profession* [title/abstract] | 98 |
| 1. allied health occupation* [title/abstract] | 23 |
| 1. allied health profession* [title/abstract] | 1783 |
| 1. assistant practitioner* [title/abstract] | 59 |
| 1. diploma nurs* [title/abstract] | 133 |
| 1. enrolled nurs* [title/abstract] | 607 |
| 1. health care assistant* [title/abstract] | 255 |
| 1. health care occupation* [title/abstract] | 61 |
| 1. “health care personnel” [title/abstract] | 2077 |
| 1. health care profession* [title/abstract] | 18327 |
| 1. “health care staff” [title/abstract] | 1015 |
| 1. health occupation* [title/abstract] | 377 |
| 1. “health personnel” [title/abstract] | 6555 |
| 1. licensed practical nurs* [title/abstract] | 544 |
| 1. “multidisciplinary care team” [title/abstract] | 106 |
| 1. nurs* [title/abstract] | 398 388 |
| 1. staff nurs* [title/abstract] | 3174 |
| 1. “patient care team” [title/abstract] | 313 |
| 1. *17 OR 18 OR 19 OR 20 OR 21 OR 22 OR 23 OR 24 OR 25 OR 26 OR 27 OR 28 OR 29 OR 30 OR 31 OR 32 OR 33 OR 34 OR 35 OR 36 OR 37 OR 38 OR 39 OR 40 OR 41* | *2061723* |

**Additional file 1.** continued

| **CINAHL Search block 3:** Qualitative studies [2017-11-16] **No items found** | |
| --- | --- |
| 1. anthropology, medical [MeSH] | 451 |
| 1. focus groups [MeSH] | 20 751 |
| 1. narration [MeSH] | 6260 |
| 1. qualitative research [MeSH] | 29165 |
| 1. ”anthropology” [all fields] | 34 634 |
| 1. ”clinical research” [all fields] | 95 460 |
| 1. ethno* [all fields] | 161 114 |
| 1. experience* [all fields] | 835 877 |
| 1. field stud* [all fields] | 12 439 |
| 1. focus group* [all fields] | 35 386 |
| 1. ”grounded theory” [all fields] | 8216 |
| 1. hermeneutic* [all fields] | 2854 |
| 1. interview* [all fields] | 301 560 |
| 1. life experience* [all fields] | 3730 |
| 1. ”narration” [all fields] | 6612 |
| 1. phenomeno* [all fields] | 193 952 |
| 1. ”qualitative research” [all fields] | 37 244 |
| 1. qualitative* [all fields] | 207 590 |
| 1. interview* [publication type] | 27 164 |
| 1. *43 OR 44 OR 45 OR 46 OR 47 OR 48 OR 49 OR 50 OR 51 OR 52 OR 53 OR 54 OR 55 OR 56 OR 57 OR 58 OR 59 OR 60 OR 61* | *1648655* |
| **CHINAL combined sets:** | |
| 1. 16 AND 42 | **10 448** |
| 1. 16 AND 61 | **13 245** |
| 1. 42 AND 61 | **238 995** |
| 1. 16 AND 42 AND 61 | **2956** |

| **ERIC Search block 1:** Intellectual developmental disorder [2017-10-29] **No items found** | |
| --- | --- |
| 1. mental retardation [DE+] | 20 526 |
| 1. pervasive developmental disorder [DE+] | 11 629 |
| 1. asperger* [AB] | 926 |
| 1. autism* [AB] | 9398 |
| 1. autistic dis* [AB] | 898 |
| 1. down syn* [AB] | 1883 |
| 1. intellectual developmental dis* [AB] | 292 |
| 1. intellectual dis* [AB] | 2696 |
| 1. learning dis* [AB] | 39178 |
| 1. mental retard* AB] | 4982 |
| 1. pervasive developmental dis* [AB] | 362 |
| 1. *1 OR 2 OR 3 OR 4 OR 5 OR 6 OR 7 OR 8 OR 9 OR 10 OR 11* | *71593* |

**Additional file 1.** continued

| **ERIC Search block 2:** Nursing [2017-10-29] **No items found** | |
| --- | --- |
| 1. allied health occupations [DE+] | 1113 |
| 1. allied health personnel [DE+] | 2160 |
| 1. nurses [DE+] | 4108 |
| 1. ”allied health care personnel” [AB] | 2 |
| 1. ”allied health care staff” [AB] | 0 |
| 1. allied health care profession* [AB] | 185 |
| 1. allied health occupation* [AB] | 271 |
| 1. allied health profession* [AB] | 554 |
| 1. assistant practitioner* [AB] | 182 |
| 1. diploma nurs* [AB] | 198 |
| 1. enrolled nurs* [AB] | 444 |
| 1. health care assistant* AB] | 359 |
| 1. health care occupation* [AB] | 829 |
| 1. “health care personnel” [AB] | 71 |
| 1. health care profession* [AB] | 3408 |
| 1. “health care staff” [AB] | 25 |
| 1. health occupation* [AB] | 3810 |
| 1. “health personnel” [AB] | 363 |
| 1. licensed practical nurs* [AB] | 245 |
| 1. “multidisciplinary care team” [AB] | 1 |
| 1. nurs* [AB] | 14 369 |
| 1. staff nurs* [AB] | 1460 |
| 1. “patient care team” [AB] | 1 |
| 1. *13 OR 14 OR 15 OR 16 17 OR 18 OR 19 OR 20 OR 21 OR 22 OR 23 OR 24 OR 25 OR 26 OR 27 OR 28 OR 29 OR 30 OR 31 OR 32 OR 33 OR 34 OR 35* | *21398* |

| **ERIC Search block 3:** Qualitative studies [2017-10-29] **No items found** | |
| --- | --- |
| 1. anthropology [DE+] | 11 256 |
| 1. experience [DE+] | 41 919 |
| 1. field studies [DE+] | 2262 |
| 1. focus groups [DE+] | 9600 |
| 1. grounded theory [DE+] | 2720 |
| 1. hermeneutics [DE+] | 1241 |
| 1. interviews [DE+] | 58 517 |
| 1. qualitative research [DE+] | 22 289 |
| 1. ”anthropology” [anywhere] | 4528 |
| 1. ”clinical research” [anywhere] | 394 |
| 1. ethno* [anywhere] | 962 |
| 1. experience* [anywhere] | 212 109 |
| 1. field stud* [anywhere] | 63 558 |
| 1. focus group* [anywhere] | 36 619 |
| 1. ”grounded theory” [anywhere] | 3367 |
| 1. hermeneutic* [anywhere] | 1597 |
| 1. interview* [anywhere] | 107 247 |
| 1. life experience* [anywhere] | 24 929 |
| 1. ”narration” [anywhere] | 2146 |
| 1. phenomeno* [anywhere] | 13 471 |
| 1. ”qualitative research” [anywhere] | 23 465 |
| 1. qualitative* [anywhere] | 46 171 |
| 1. *37 OR 38 OR 39 OR 40 OR 41 OR 42 OR 43 OR 44 OR 45 OR 46 OR 47 OR 48 OR 49 OR 50 OR 51 OR 52 OR 53 OR 54 OR 55 OR 56 OR 57 OR 58* | *384623* |
| **ERIC combined sets:** | |
| 1. 12 AND 36 | **972** |
| 1. 12 AND 59 | **20 760** |
| 1. 36 AND 59 | **7076** |
| 1. 12 AND 36 AND 59 | **412** |

**Additional file 1.** continued

| **PsycINFO Search block 1:** Intellectual developmental disorder [2017-10-30] **No items found** | |
| --- | --- |
| 1. autism spectrum disorders [SU.EXACT] | 33747 |
| 1. down´s syndrome [SU.EXACT] | 5575 |
| 1. intellectual development disorder [SU.EXACT] | 41303 |
| 1. learning disorders [SU.EXACT] | 31 810 |
| 1. learning disabilities [SU.EXACT] | 25 693 |
| 1. asperger* [AB] | 3154 |
| 1. autism* [AB] | 31 797 |
| 1. autistic dis* [AB] | 4503 |
| 1. down syn* [AB] | 7786 |
| 1. intellectual developmental dis* [AB] | 1205 |
| 1. intellectual dis* [AB] | 9201 |
| 1. learning dis* [AB] | 49601 |
| 1. mental retard* AB] | 15 147 |
| 1. pervasive developmental dis* [AB] | 2096 |
| 1. *1 OR 2 OR 3 OR 4 OR 5 OR 6 OR 7 OR 8 OR 9 OR 10 OR 11 OR 12 OR 13 OR 14 OR 15* | *160884* |

| **PsycINFO Search block 2:** Nursing [2017-10-30] **No items found** | |
| --- | --- |
| 1. allied health personnel [SU.EXACT+] | 4407 |
| 1. health personnel [SU.EXACT+] | 118 128 |
| 1. ”allied health care personnel” [AB] | 0 |
| 1. ”allied health care staff” [AB] | 1 |
| 1. allied health care profession* [AB] | 574 |
| 1. allied health occupation* [AB] | 201 |
| 1. allied health profession* [AB] | 1261 |
| 1. assistant practitioner* [AB] | 434 |
| 1. diploma nurs* [AB] | 188 |
| 1. enrolled nurs* [AB] | 1279 |
| 1. health care assistant* AB] | 792 |
| 1. health care occupation* [AB] | 2962 |
| 1. “health care personnel” [AB] | 2217 |
| 1. health care profession* [AB] | 27 639 |
| 1. “health care staff” [AB] | 369 |
| 1. health occupation* [AB] | 11 831 |
| 1. “health personnel” [AB] | 669 |
| 1. licensed practical nurs* [AB] | 213 |
| 1. “multidisciplinary care team” [AB] | 17 |
| 1. nurs* [AB] | 77 808 |
| 1. staff nurs* [AB] | 9988 |
| 1. “patient care team” [AB] | 22 |
| 1. *17 OR 18 OR 19 OR 20 OR 21 OR 22 OR 23 OR 24 OR 25 OR 26 OR 27 OR 28 OR 29 OR 30 OR 31 OR 32 OR 33 OR 34 OR 35 OR 36 OR 37* | *194224* |

**Additional file 1.** continued

| **PsycINFO Search block 3:** Qualitative studies [2017-10-30] **No items found** | |
| --- | --- |
| 1. anthropology [SU.EXACT+] | 5485 |
| 1. ethnography [SU.EXACT+] | 7348 |
| 1. ethnology [SU.EXACT+] | 1952 |
| 1. life experiences [SU.EXACT+] | 25 406 |
| 1. hermeneutics [SU.EXACT+] | 1862 |
| 1. interviews [SU.EXACT+] | 11 925 |
| 1. narratives [SU.EXACT+] | 16 422 |
| 1. phenomenology [SU.EXACT+] | 11 561 |
| 1. qualitative research [SU.EXACT+] | 7176 |
| 1. ”anthropology” [anywhere] | 29 909 |
| 1. ”clinical research” [anywhere] | 22 661 |
| 1. ethno* [anywhere] | 29 905 |
| 1. experience* [anywhere] | 545 918 |
| 1. field stud* [anywhere] | 161 278 |
| 1. focus group* [anywhere] | 83 132 |
| 1. ”grounded theory” [anywhere] | 12 031 |
| 1. hermeneutic* [anywhere] | 6143 |
| 1. interview* [anywhere] | 401 112 |
| 1. life experience* [anywhere] | 112 133 |
| 1. ”narration” [anywhere] | 1674 |
| 1. phenomeno* [anywhere] | 79 056 |
| 1. ”qualitative research” [anywhere] | 19 621 |
| 1. qualitative* [anywhere] | 230 511 |
| 1. *37 OR 38 OR 39 OR 40 OR 41 OR 42 OR 43 OR 44 OR 45 OR 46 OR 47 OR 48 OR 49 OR 50 OR 51 OR 52 OR 53 OR 54 OR 55 OR 56 OR 57 OR 58* | *1175751* |
| **PsycINFO Combined sets:** | |
| 1. 16 AND 39 | **5025** |
| 1. 16 AND 63 | **44 627** |
| 1. 39 AND 63 | **78 813** |
| 1. 12 AND 36 AND 63 | **4049** |
