# Supplementary material for: Nurses’ experiences of caring for patients with intellectual developmental disorders: a systematic review using a meta-ethnographic approach
Source: BMC Nurs. 2018 Dec 3;17:51. doi: 10.1186/s12912-018-0316-9 (PMC6276187; doi:10.1186/s12912-018-0316-9)
Supplement: Supplementary file 3 — Studies excluded after full text reading. (DOCX 22 kb) [file 12912_2018_316_MOESM3_ESM.docx]

**Additional file 3.** Excluded studies after full text reading

| **Author, year** | **Excluded sift 1-3** | **Reason for exclusion** |
| --- | --- | --- |
| Bekkema *et al.,* 2015 | 1 | Do not meet review aim |
| Brown *et al.,* 2003 | 1 | Not accessible |
| Butrimaviciute & Grieve, 2014 | 1 | Do not meet review aim |
| Capri & Buckle, 2015 | 1 | Do not meet review aim |
| Cardol *et al.*, 2012 | 1 | Do not meet review aim |
| Cartlidge & Read, 2010 | 1 | Do not meet review aim |
| Donner *et al.*, 2010 | 1 | Do not meet review aim |
| Drozd & Clinch 2015 | 2 | Do not meet inclusion criteria; Data is based on a questionnaire |
| Fish, 2000 | 2 | Do not meet review aim |
| Findlay *et* al., 2015 | 1 | Do not meet review aim |
| Forster & Iacono, 2008 | 1 | Do not meet review aim |
| Galvin & Timmins, 2012 | 2 | Do not meet review aim |
| Gibbs *et al*., 2008 | 1 | Do not meet review aim |
| Handley & Hutchinson, 2013 | 1 | Do not meet review aim |
| Hawkins *et al.*, 2005 | 1 | Do not meet review aim |
| Hellzen & Asplund, 2002 | 3 | Do not meet review aim |
| Hutchinson *et al.*, 2012 | 1 | Do not meet review aim |
| Hutchinson & Stenfert Kroese, 2016 | 2 | Do not meet review aim |
| Iacono *et al.*, 2014 | 1 | Do not meet review aim |
| Isherwood *et al.*, 2006 | 2 | Do not meet inclusion criteria; Data is based on a questionnaire |
| Jones *et al.*, 2007 | 1 | Do not meet review aim |
| Jones, 2014 | 1 | Do not meet review aim |
| Lloyd & Coulson, 2014 | 2 | Do not meet review aim |
| Lloyd & Coulson, 2014 | 1 | Do not meet review aim |
| Lovell *et al.*, 2014 | 1 | Do not meet review aim |
| Lovell & Skellern 2013 | 1 | Do not meet review aim |
| Lundström *et al.*, 2007 | 1 | Do not meet review aim |
| Lunsky *et al.*, 2008 | 2 | Do not meet review aim |
| McCarron *et al.*, 2010 | 1 | Do not meet review aim |
| McEvoy *et al.*, 2010 | 1 | Do not meet review aim |
| McIlfatrick *et al*., 2011 | 1 | Do not meet review aim |
| McLaughlin *et al.*, 2007 | 2 | Do not meet review aim |
| Mérineau-Côté & Morin, 2014 | 1 | Do not meet review aim |
| Pimentel & Ryan, 1996 | 2 | Do not meet review aim |
| Raczka, 2005 | 1 | Do not meet review aim |
| Ryan *et al*., 2011 | 1 | Do not meet review aim |
| Sandhu *et al*., 2012 | 1 | Do not meet review aim |
| Sowney & Barr, 2007 | 1 | Do not meet review aim |
| Taggart *et al*., 2010 | 1 | Do not meet review aim |
| Taggart *et al*., 2011 | 1 | Do not meet review aim |
| Taylor, J. & Trout, S. (2013). | 2 | Do not meet review aim |
| Thornton, 1996 | 2 | Do not meet review aim |
| Todd, (2013 | 1 | Do not meet review aim |
| Warfield *et al*., 2015 | 1 | Do not meet review aim |
| Wiese *et al*., 2012 | 2 | Do not meet review aim |
| Wiese *et al*., 2013 | 1 | Do not meet review aim |
| Weiss *et al*., 2009 | 1 | Do not meet review aim |
| Wilkinson *et al*., 2012 | 1 | Do not meet review aim |
| Willis *et al*., 2010 | 1 | Do not meet review aim |
|  |  |  |
